# Association of Prostate Multiparametric MRI Parameters with Gleason Score: A Fusion Biopsy-Based Correlation

**DOI:** 10.2174/0115734056436931260214191215

**Published:** 2026-03-11

**Authors:** Davut Ünsal Capkan, Mehmet Hamdi Sahan

**Affiliations:** 1 Department of Radiology, Gaziantep Private Medicalpoint Hospital, Gaziantep, Turkiye; 2 Department of Radiology, Faculty of Medicine, Gaziantep University, Gaziantep, Turkiye

**Keywords:** Biopsy, Diffusion magnetic resonance imaging, Magnetic resonance imaging, Pathology, Prostatic neoplasms, Prostate-specific antigen

## Abstract

**Introduction:**

This study aimed to investigate the relationship between imaging parameters obtained from Multiparametric Magnetic Resonance İmaging (MP-MRI) and Gleason Scores (GS) derived from targeted prostate biopsies performed using MRI/Ultrasound (US) fusion guidance.

**Materials and Methods:**

A total of 120 consecutive patients who underwent 1.5T MP-MRI followed by MRI/US fusion-guided prostate biopsy were included. In total, 202 MRI-identified targets were sampled. For each lesion, anatomical location, Prostate-Specific Antigen (PSA), prostate volume, Prostate Density (PD), apparent diffusion Coefficient (ADC) values, neurovascular bundle invasion, and PI-RADS v2.1 score were recorded. GS results were statistically compared with imaging and clinical parameters. Pearson’s correlation was calculated to investigate the associations, and the diagnostic ability of ADC values was evaluated by Receiver Operating Characteristic (ROC) analysis.

**Results:**

Of the 202 targets, 138 (68.3%) were benign, 35 (17.3%) had GS 3+4 (low-grade), and 29 (14.4%) had GS ≥4+3 (high-grade) malignancy. ADC values were negatively correlated with age, PSA, PD, and PI-RADS score. Most lesions were located in the peripheral zone, particularly in the mid-gland and apical regions. Malignant lesions were frequently found in the peripheral zone and right mid-gland. ADC values showed strong diagnostic performance, with cut-offs of 0.78 ×10^−3^ mm^2^/s for low-grade (sensitivity: 79.69%, specificity: 86.21%) and 0.70 ×10^−3^mm^2^/s for high-grade malignancies (sensitivity: 92.03%, specificity: 92.49%).

**Discussion:**

This echoes previous reports indicating that ADC value negatively correlated with GS, supporting the application of MP-MRI-derived parameters in the assessment of prostate cancer.

**Conclusion:**

Correlation of MP-MRI-derived imaging parameters with histopathology results allows meaningful exploitation in clinical decision-making in prostate cancer diagnosis. Both the PI-RADS score and the ADC value were validated as reliable predictors of tumor aggressiveness. Although MRI-targeted fusion biopsies have gained more and more popularity in clinical practice, based on our data, no conclusion can be drawn as to their effect in decreasing the number of unnecessary prostate biopsies.

## 1 INTRODUCTION

Prostate cancer is still one of the most common cancers in the world and the second most frequently diagnosed cancer in men [1]. Proper detection and risk classification are important for recommending suitable treatment and preventing over- or under-diagnosis. However, conventional imaging techniques, such as Transrectal Ultrasound (TRUS), have demonstrated poor capability to identify prostate cancer lesions, especially significant ones [2].

In recent years, Multiparametric Magnetic Resonance İmaging (MP-MRI) has emerged as a non-invasive and effective imaging method for evaluating prostate cancer [2, 3]. The combined use of T2-weighted imaging, Diffusion-Weighted İmaging (DWI), Apparent Diffusion Coefficient (ADC) mapping, and dynamic contrast-enhanced imaging techniques in MP-MRI facilitates the detection, localization, and staging of lesions [2-4]. These imaging parameters are an integral part of the Prostate Imaging Reporting and Data System (PI-RADS), which is designed to standardize MP-MRI interpretation and guide biopsy decisions. Especially in peripheral lesions, the switch from PI-RADS 3 to PI-RADS 4 or 5, which affects biopsy recommendations, largely depends on DWI and ADC characteristics [5].

Despite the utility of PI-RADS, its semi-quantitative and somewhat subjective assessment criteria, especially in DWI and ADC interpretation, may introduce interreader variability and diagnostic inconsistency [2]. To this end, quantitative MP-MRI parameters, for example ADC values, have recently been suggested in order to offer objective imaging biomarkers that show better correlation with tumor aggressiveness [6, 7]. Pathological grading of prostate cancer, most commonly with the Gleason Scoring (GS) system, is still considered the best predictor of tumor aggressiveness and the cornerstone in management [8]. The integration of MRI/ultrasound (US) fusion biopsy, which combines MP-MRI-based lesion targeting with real-time ultrasound guidance, has allowed for more accurate sampling of suspicious lesions compared to traditional systematic TRUS-guided biopsies [3, 9].

The aim of this study was to evaluate the relationship between imaging parameters obtained with MP-MRI and GS obtained from target prostate biopsies performed under MR/US fusion guidance. Establishing a statistically supported correlation between MRI findings and histopathology may further clarify the role of MP-MRI in prostate cancer diagnostics and improve patient selection for biopsy and treatment.

## 2 MATERIAL AND METHODS

### 2.1 Study Population

**Fig. (1):**
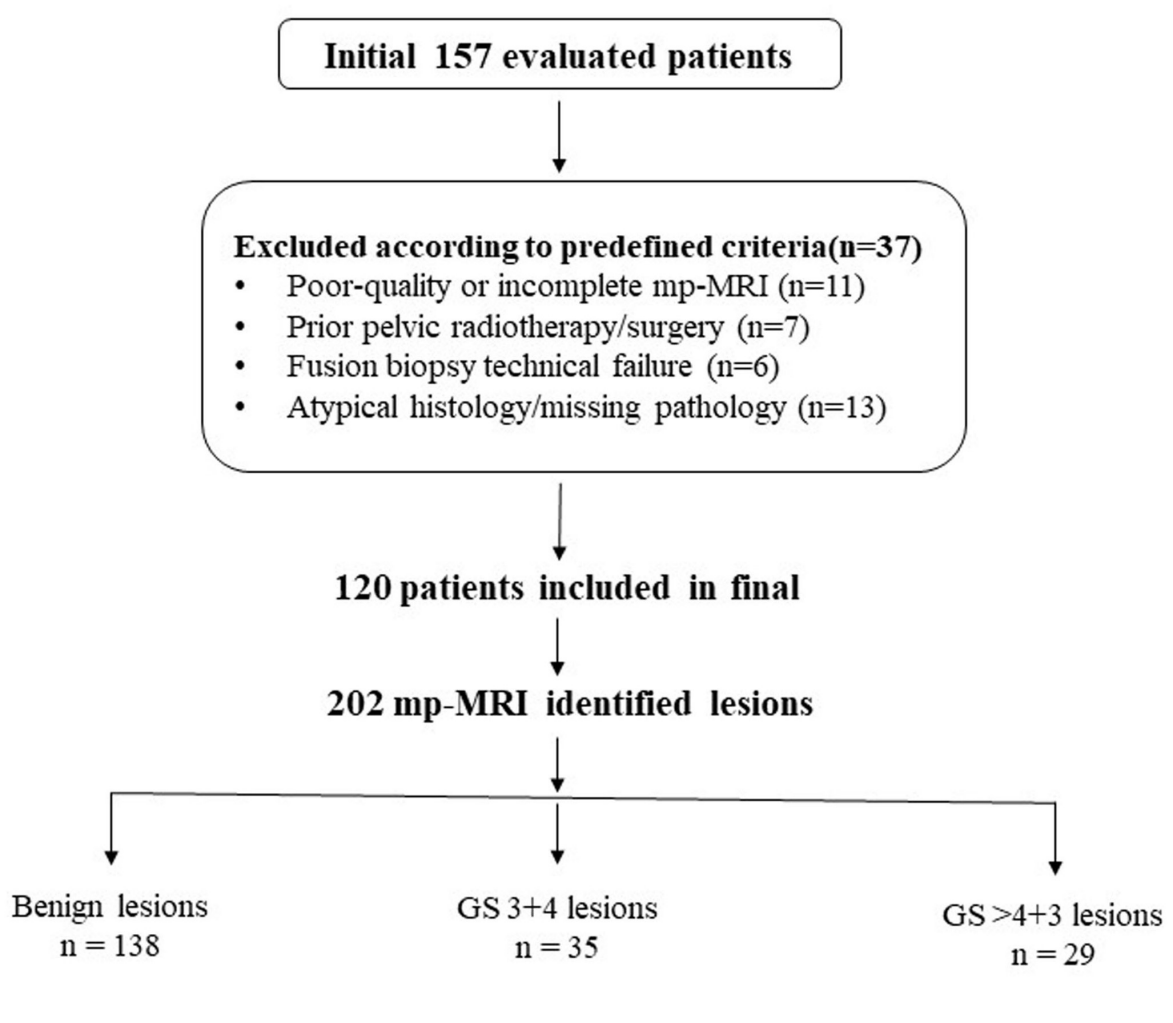
Study population flow diagram.

**Fig. (2):**
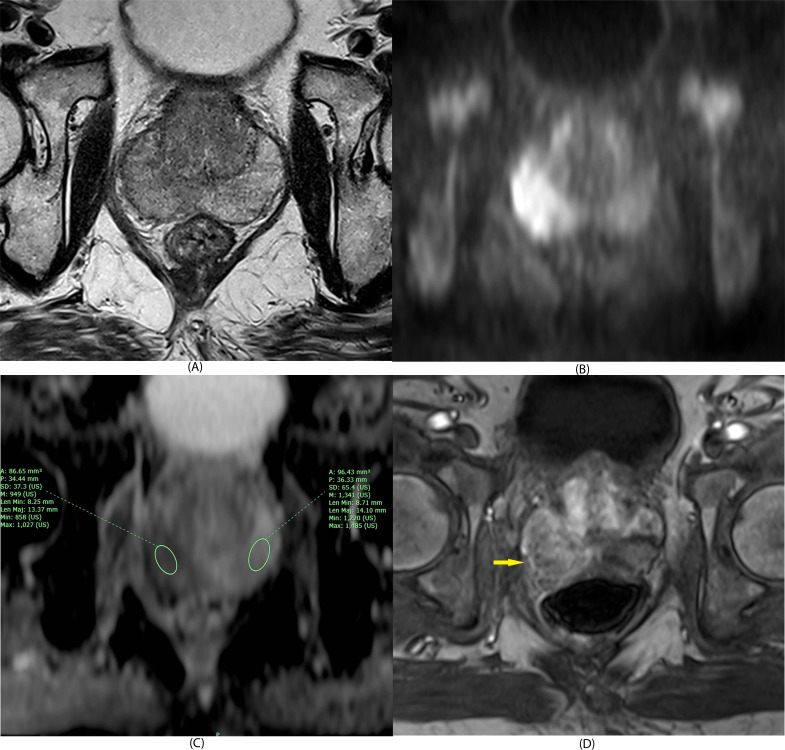
A 65-year-old patient with a target lesion located at the right peripheral zone midgland level. Axial T2-weighted image (**A**), diffusion-weighted image (**B**), ADC map with measurement (**C**), and axial contrast-enhanced image (**D**) are shown (yellow arrow). ADC: apparent diffusion coefficient.

Between January 2022 and November 2024, a total of 120 consecutive patients who were referred with clinical suspicion of prostate cancer and underwent 1.5 Tesla (T) MP-MRI followed by MRI/US fusion prostate biopsy were included in this retrospective study (Fig. **1**). All participants were aged between 35-80 years. A total of 202 MP-MRI identified targets were sampled (Fig. **2A**-**D**).

The patient's data were obtained from the electronic medical record system of the institution as well as radiology information systems, including the picture archiving and communication system, pathology databases, and laboratory results. To protect patient confidentiality, all data were anonymized before analysis.

The location of each lesion was documented on the basis of MP-MRI findings [2]. Prostate volume was calculated from T2-weighted images [10], and total serum Prostate-Specific Antigen (PSA) levels were obtained from the hospital laboratory. Prostate Density (PD) = PSA/prostate volume [11]. The mean ADC values (mm^2^/s × 10^−3^) were manually measured in a freehand-drawn Region Of İnterest (ROI) in every lesion (Fig. **2c**) [6]. Also, an MRI was performed to evaluate for neurovascular bundle invasion and pelvic lymph node metastases. Lesions were assessed with PI-RADS, version 2.1, and histopathology and GS of the respective lesions.

All subjects in this research were Turkish. The Turkish people are said to be a genetically homogeneous group, which reduces the biological variance and enables more standardized evaluation of quantitative measures such as ADC maps.

### 2.2 Exclusion Criteria

The following exclusion criteria were used for data quality and homogeneity: (1) incomplete or low quality MP-MRI information, (2) previous radical prostatectomy and/or pelvic radiotherapy, (3) technical limitations during the fused biopsy process that precluded accurate lesion sampling, (4) ductal or other unusual growth patterns or histological subtypes, and (5) no histopathological findings. Patients were systematically evaluated against these criteria. Furthermore, any patients who were later found to meet these exclusion criteria were excluded from the final analysis (Fig. **1**).

### 2.3 MP-MRI and Biopsy Protocol

A 1.5T MRI scanner (Symphony, Siemens, Germany) equipped with 16-channel phased-array body coils was used. MP-MRI protocol included anatomical T2-weighted and T1-weighted imaging, DWI, and dynamic contrast-enhanced imaging. T2-weighted images were acquired in the axial and sagittal planes using the following parameters: Repetition Time (TR) 4000-6000 ms, Echo Time (TE) 90-120 ms, matrix size 320 × 256, Field Of View (FOV) 16-20 cm, slice thickness 3 mm, and interslice gap 0.3 mm. T1-weighted images were acquired primarily for anatomical correlation following contrast administration (TR: 500-700 ms, TE: 10-15 ms).

DWI was obtained in the axial plane using b-values of 0, 800, and 1500 s/mm^2^. DWI parameters included: TR 3000-4000 ms, minimum TE, matrix 128 × 128, FOV 20 cm, and slice thickness 3 mm. An echo planar imaging sequence was used for DWI acquisition. For dynamic contrast-enhanced imaging, all patients were scanned in the axial plane using a 3D T1-weighted gradient-echo sequence, with the following parameters: TR 4.5-6.0 ms, TE 1.5-2.0 ms, flip angle 12°, matrix 256 × 192, FOV 20 cm, and slice thickness 3 mm. All patients received an intravenous injection of gadoterate meglumine at a dose of 0.1 ml/kg, administered at a rate of 3 ml/s. The temporal resolution for dynamic contrast-enhanced imaging was set at 15 seconds.

All MP-MRI examinations were processed on a Syngo *via* VB30 workstation (Siemens Healthineers, Germany). ADC maps were automatically generated by the system, and ROI-based ADC measurements were performed manually on the workstation by an interventional radiologist with 7 years of experience (D.Ü.Ç.). Multiplanar reconstructions and lesion measurements were created using the same platform.

Dynamic contrast-enhanced sequences were acquired after intravenous administration of 0.1 mmol/kg gadoterate meglumine (Dotarem^®^, Guerbet, France) at a rate of 3 mL/s, followed by a 20 mL saline flush.

Fusion biopsy was performed using the bkFusion MRI–US fusion platform (BK Medical, USA; https://www.bkmedical.com/bkfusion-mr-fusion-biopsy-6/), which allows real-time superimposition of axial T2-weighted MRI images onto transrectal ultrasound images. The technique was based on the approach described by Sonn *et al*. [9].

Sedoanalgesia was performed using intravenous midazolam (0.03–0.05mg/kg) and fentanyl (1–2 μg/kg), administered by an anesthesiologist to provide conscious sedation. For all procedures, either an 18-gauge or 16-gauge core biopsy needle (*e.g*., Bard Max-Core or similar device) was used. Three core biopsies were obtained from each ROI. In addition, a systematic 12-core biopsy was performed for each patient. Further technical details of the biopsy procedure have been described previously [12, 13].

The pathology results of the targeted lesions were categorized as benign, low-grade malignancy (GS 3+4), or high-grade malignancy (GS ≥4+3).

### 2.4 Statistical Analysis

Statistical analyses were performed using SPSS version 20 software. Data are expressed as mean ± Standard Deviation (SD) or median (range), as appropriate. The normality of variables was tested, and equal variance was assumed. Comparisons between groups were made using one-way ANOVA for continuous normally distributed variables and the chi-square test for categorical variables.

Stratified analyses were conducted by GS categories (benign, GS 3+4, GS >4+3) to account for possible confounders. Further, Pearson correlation analysis was applied to analyze the correlation between continuous variables, with adjustment of confounders by stratification using clinical characteristics, including age, PSA, and prostate volume. To control for Type I error, the Bonferroni correction was used when multiple comparisons were conducted.

Receiver Operating Characteristic (ROC) analyses were conducted to find ADC cut-off values that most effectively separate malignant (both low and high GS) from benign lesions with the best sensitivity and specificity. Based on these optimal cut-off values, we calculated the sensitivity, specificity, Positive Predictive Value (PPV), and Negative Predictive Value (NPV). ROC analyses were performed with SPSS version 20.0.

*p* < 0.05 was considered statistically significant.

#### 2.4.1 Sample Size

The a priori sample size calculation suggested 53 participants per group were necessary to detect a medium effect size (Cohen's d = 0.5) with α = 0.10 and 1-β = 0.90. There were 60 subjects per group in our study (n = 120), which was sufficient to provide enough statistical power. The computation was carried out with G*Power 3.1.

## 3 RESULTS

The demographic and clinical characteristics of the 120 patients are shown in Table **1**.

MRI/US fusion biopsy was performed to assess 202 targeted prostate lesions. Of these 202 lesions, 138 (68.3%) were benign on histopathology, 35 (17.3%) were GS 3+4, and 29 (14.4%) were GS >4+3, representing high-grade malignancy. Consistent with current clinical guidelines recommending clinical significance of prostate cancer as GS≥3+4 [3], 64 lesions (31.7%) were clinically significant. These results demonstrate that MR/US fusion biopsy provides a high detection rate for clinically significant prostate cancer, and yet has the ability to separate indolent or benign lesions.

Table **2** compares Age, PSA, PI-RADS, and MP-MRI findings by GS groups.

### 3.1 Correlation of ADC with Clinical Parameters

A statistically significant inverse correlation was found between the ADC values and age, PSA level, PD, and PI-RADS category. In contrast, a significant positive correlation was observed with prostate volume (*p*< 0.05 for all). These relationships are shown in Table **3**.

### 3.2 Diagnostic Performance of ADC Values

ROC curve analyses were conducted to calculate optimal ADC cut-off values that could provide the best separation between malignant and benign tumors with the highest sensitivity and specificity.

ROC analysis revealed that ADC values could distinguish between benign and malignant lesions with different cutoff points according to the Gleason grades:

In low-grade malignancy (GS 3+4), sensitivity > specificity (79.69% *vs*. 86.21%) was obtained at an ADC cut-off value of 0.78 × 10^−3^ mm^2^/s.

For high-grade malignancy (GS >4+3), the ADC cut-off value of 0.70 × 10^−3^ mm^2^/s provided a sensitivity of 92.03% and specificity of 92.49%.

**Fig. (3):**
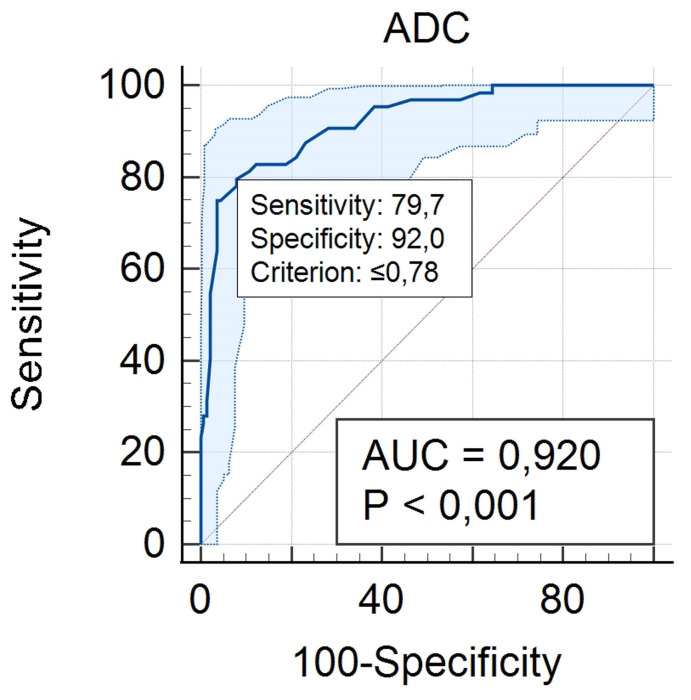
Receiver Operating Characteristic (ROC) curve demonstrating the diagnostic performance of ADC values (mm^2^/s) for distinguishing low-grade malignant lesions (GS 3+4) from benign targets. The x-axis represents the false-positive rate (1 − specificity), and the y-axis represents the true-positive rate (sensitivity). The Area Under The Curve (AUC) reflects the overall discriminative ability of ADC values. The optimal ADC cut-off point for low-grade malignancy is indicated on the curve.

**Fig. (4):**
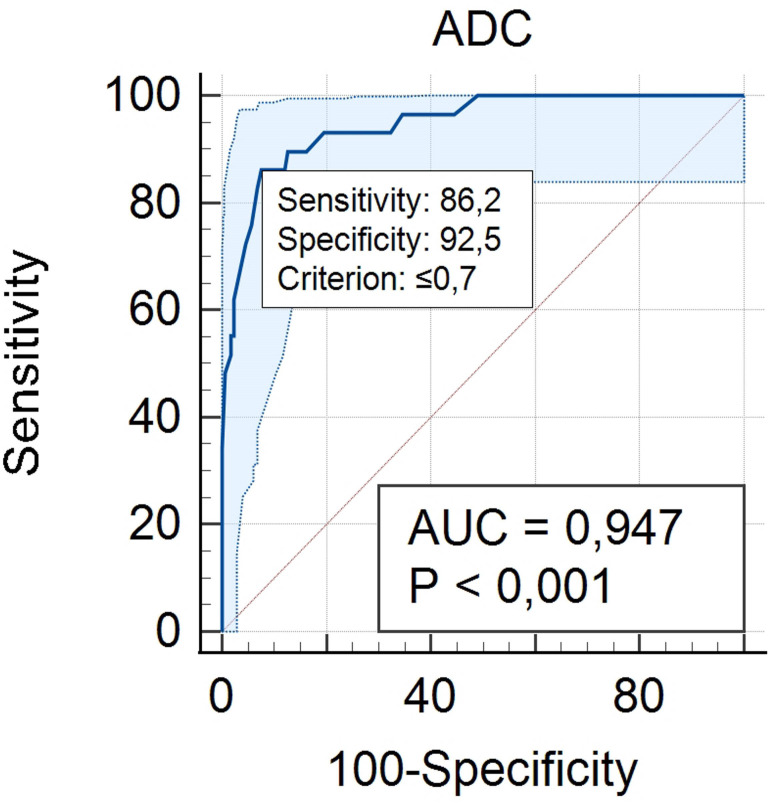
Receiver Operating Characteristic (ROC) curve demonstrating the diagnostic performance of ADC values (mm^2^/s) for distinguishing high-grade malignant lesions (GS >4+3) from benign targets. The x-axis represents the false-positive rate (1 − specificity), and the y-axis represents the true-positive rate (sensitivity). The Area Under The Curve (AUC) reflects the overall discriminative ability of ADC values. The optimal ADC cut-off point for high-grade malignancy is indicated on the curve.

These findings indicate high diagnostic accuracy of ADC values in predicting tumor aggressiveness (Table **4**, Figs. **3** and **4**).

### 3.3 Lesion Zonal and Anatomical Distribution

When the zonal distribution of lesions was assessed:

138 benign lesions were primarily located in the peripheral zone (n=118, 85.5%), with the remaining 20 lesions (14.5%) found in the transitional zone.

Among GS 3+4 lesions, 32 (91.4%) were located in the peripheral zone, and 3 (8.6%) in the transitional zone.

Of the GS >4+3 lesions, 26 (90%) were in the peripheral zone, while 3 (10%) were in the transitional zone.

Anatomically, lesions were most frequently located in the middle gland (118/202, 58.4%), followed by the apex (48/202, 23.79%) and base (36/202, 17.8%) (Table **5**).

### 3.4 Laterality of Lesions

Benign lesions were almost equally distributed between the two lobes, with 70 lesions (50.7%) on the right and 68 (49.3%) on the left. However, malignant lesions exhibited a tendency to localize more frequently on the right side:

GS 3+4 lesions: 24 (68.5%) right, 11 (31.5%) left.

GS >4+3 lesions: 19 (65.5%) right, 10 (34.5%) left.

Malignant lesions were predominantly located in the right peripheral zone, particularly in the middle gland, supporting the regional predilection for clinically significant prostate cancer (Table **5**).

Histopathological results and biopsy positivity rates of target lesions are presented in Table **6**.

The distribution of lesions, malignancy, and biopsy core positivity according to PI-RADS categories is presented in Table **7**.

## 4 DISCUSSION

MP-MRI has demonstrated high sensitivity in distinguishing prostate cancer from benign prostatic conditions. In this study, MP-MRI parameters such as age, PSA, prostate volume, PD, PI-RADS score, and especially ADC values, showed a statistically significant correlation with histopathological results obtained from MR/US fusion-guided prostate biopsies (Table **3**). Among these, ADC values emerged as robust imaging biomarkers for predicting the degree of malignancy, particularly differentiating high-grade disease (Table **4**, Figs. **3** and **4**).

The risk of prostate cancer increases with age [14]. While many studies link advanced age to more aggressive disease, some have reported that age alone does not always correlate directly with GS or tumor grade [15-17]. In imaging, the influence of age is subtle, though age-related prostatic fibrosis may lead to slightly reduced ADC values in older patients [16, 17].

PSA remains a widely used biomarker in prostate cancer screening, despite its limitations in specificity. Elevated PSA may result from benign prostatic hyperplasia or prostatitis [16]. However, higher PSA levels are often associated with PI-RADS 4 and 5 lesions and, accordingly, higher histological grades [16, 18, 19]. The current study supports this, as increasing PI-RADS scores were associated with a higher likelihood of clinically significant prostate cancer (GS >3+4) (Table **3**), consistent with existing literature [17, 18].

Prostate volume plays an important role in interpreting PSA values. Larger prostates may naturally produce more PSA, complicating interpretation. Therefore, PD, calculated as PSA/prostate volume, is considered a more accurate predictor of malignancy [11]. PD values greater than 0.15 ng/mL/cc were reported to be associated with an increased risk of clinically meaningful disease [11]. In our study, an inverse relation was observed between prostate volume and high-grade malignancy, whereas elevated PD values were linked to higher GS (Tables **2** and **3**).

DWI has an important role in the noninvasive detection and characterization of PCa. One of its important quantifiable parameters, ADC, is a measure of the diffusivity of water molecules in tissue, and is inversely correlated to cellular density, a hallmark of malignant transformation [20-22]. In agreement with the progressive increase in tumor cellularity and architectural distortion associated with high-grade disease [20-23], several studies have uniformly found that as GS increases, ADC values decrease.

Tumor cellularity is a significant contributor to grade in prostate cancer. Studies such as by Hambrock *et al.* [23], utilizing 3.0 Tesla MRI, validated that ADC values distinctly separate low -, intermediate -, and high-grade disease. In a similar observation, Kim *et al*. [17] showed a mean ADC value of 0.741 ± 0.164 × 10-3 mm2/sec for GS 7 lesions, which is quite consistent with the values of our study. Salami *et al.* [24] also noted that larger, more aggressive prostate cancers had lower ADC values.

Apart from qualitative relationships, some studies also suggested numeric values of ADC cut-offs to predict malignancy. For example, Virendra Kumar *et al.* [25] reported a cut-off of 1.17 × 10^−3^ mm^2^/sec in the peripheral and central zones with 73% sensitivity and 74% specificity. Other reports state a higher range for ADC sensitivity (29-94%) and specificity (39-100%) [17, 26, 27], and meta-analyses show that variability overall is low [21, 27, 28].

Similar to the literature, our results also showed that the ADC value held a high predictive value for malignancy (Table **4**). ROC curve analysis revealed that the lower ADC cut-off value was useful for differentiating both low (GS 3+4) and high (GS >4+3) grade PCa with high sensitivity and specificity (Figs. **3** and **4**). These findings suggest that ADC may serve as a useful, practical, and quantifiable marker of tumor aggressiveness.

Previous reports on MP-MRI are therefore an integral basis for our results. Sonn *et al*. [9, 29, 30] also found that MRI US fusion biopsy increased detection of clinically significant prostate cancer, particularly in PI-RADS 4–5 lesions, similar to our finding that higher PI-RADS scores correlate with higher GS categories. Supporting findings were observed by Kim *et al.* [17] and Hambrock *et al.* [23] showing the reverse correlation of ADC and tumor aggressiveness, which is reproduced in our cohort and strengthens the use of ADC as a consistent quantitative marker for different MRI machine types, including 1.5 T scanners. Although a higher ADC threshold for malignancy (1.17 × 10^−3^ mm^2^/s) has been suggested by Kumar *et al.* [25], the lower optimal cut-off value reveals the known variability between different institutions, presumably due to the different machine vendor and acquisition protocols and the characteristics of the patient population. Salami *et al*. [24] reported that ADC combined with lesion volume improves risk stratification, and although lesion volume was not assessed in our study, the strong standalone predictive value of ADC aligns with their observations. Furthermore, Bengtsson *et al.* [31] confirmed high cross-field reproducibility of ADC between 1.5T and 3T systems, supporting the applicability of our findings despite using 1.5T imaging. Consistency with Asian MP-MRI studies by Dai *et al.* [19] and Han *et al*. [18] indicates that PI-RADS scoring and ADC metrics maintain diagnostic performance across diverse ethnic backgrounds. Overall, these comparisons indicate that the performance of MP-MRI parameters, particularly ADC, is highly consistent with previous reports and strengthens the evidence supporting their role in guiding risk stratification and biopsy decision-making in prostate cancer.

With regard to patient demographics, our study population consisted entirely of individuals of Turkish ethnic origin, a population considered relatively genetically homogeneous, as described in the Methods section. This homogeneity reduces biological variability and allows a more homogeneous assessment of quantitative MRI parameters like ADC. The lack of racial and ethnic diversity limits generalizability to other populations but also reduces endogenous variability and enhances the ability to detect differences within cohorts. Notably, the results of our multi-ethnic cohort were in line with those of the report by Sonn *et al*. [9] and with the more recent single-center studies from Asia and Europe [18, 19], indicating that several important MP-MRI performance parameters, including the diagnostic value of ADC, demonstrated good generalizability to different ethnic and racial groups. Taken together, these results indicate that our findings are in line with the existing MP-MRI literature and further support the classification according to GS with MRI/US fusion biopsy as the reference standard.

Our study supports the utility of MP-MRI parameters, and specifically ADC, as a useful tool for clinical decisions. The stronger association between ADC values and histopathological GS indicates that this imaging biomarker could be incorporated into routine diagnostic procedures.

Combining the MP-MRI results with clinical parameters, including PSA, PD, and PI-RADS score, can significantly enhance the prediction of patients with a high possibility of harboring targetable lesions, thus facilitating more accurate candidate selection for targeted biopsy, AS, or definitive treatment. Specifically, the negative correlation between ADC and GS in our study is consistent with previous findings reporting that MP-MRI has a high sensitivity for the detection of clinically significant disease and high-risk patients who are potential candidates for more aggressive diagnostic or therapeutic strategies.

Although radical prostatectomy specimens were not available in our cohort, fusion-guided biopsies on the basis of mpMRI findings yielded meaningful histopathological information. This strategy, already supported by studies such as those of Dell’Oglio *et al.* [12] and Stabile *et al.* [13], is being progressively recognized as the clinical standard for prostate cancer diagnosis, especially in the case of patients who do not undergo immediate surgery.

Furthermore, the benefits of MRI-targeted biopsy have not eliminated the importance of systematic biopsy, so that several studies keep reiterating a systematic approach in addition to targeted biopsies in order not to miss any clinically significant cancers [29, 30].

All the MP-MRI scans in our study were conducted using a 1.5 T system, which may have an impact on the absolute value of ADC. Some literature reports that the 3T system provides superior spatial resolution and signal-to-noise ratio, which may lead to better lesion detectability. However, ADC values were shown to be relatively similar on 1.5T and 3T systems with small differences that would not influence clinical reading [31]. A multicentre study by Bengtsson *et al.* [31] verified the high correlation of ADC values between the two MRI field strengths, underscoring that our findings are both comparable and reproducible.

## 5 LIMITATIONS AND FUTURE DIRECTIONS

There are several limitations to this study. First, the retrospective design of the study may have led to inherent biases. Moreover, the small number of high-grade (GS >4+3) lesions may affect the statistical power, and these findings must be interpreted with caution. Another limitation is that we did not assess reproducibility. Test-retest measurements were not obtained, nor was analysis of intra- or inter-observer agreement for ADC measurements and PI-RADS scoring. All interpretations of MRI and performance of the fusion biopsy were carried out by one radiologist, which eliminates variability in technique and may facilitate consistency in obtaining images and performing fusion biopsy, but limits generalizability to other radiologists. Our reference standard was limited to biopsy results and did not include radical prostatectomy specimens, thereby limiting complete histopathological correlation. Furthermore, no direct comparison was conducted between benign target biopsies and different GS of clinically insignificant prostate cancers. It is also worth mentioning that absolute ADC values may differ between institutions related to varying MRI scanners, imaging sequences, and b-values, among others. Even so, our study contributes to the literature with comprehensive results and clinical comparisons based on GS. In the future, we suggest large-scale prospective studies to investigate inter-observer variability in MRI interpretation and biopsy targeting, better compare various histological subtypes, and investigate the prognostic significance of MRI parameters, as well as to study the effect of fusion biopsy on patient management.

## CONCLUSION

This study shows statistically significant correlations between parameters derived from MP-MRI and the histopathologic GS of targeted lesions in the prostate. Lesions were mainly situated in the peripheral zone, mostly in the mid-gland and apex. The PI-RADS score and ADC value were good predictors of tumor aggressiveness, and PD was more strongly associated with malignancy than PSA. The significant negative correlations between ADC values and PI-RADS score, PSA, and PD highlight the supplementary function of imaging in prostate cancer evaluation. While confirming previous knowledge on the value of MP-MRI and DWI, the study adds detail by stratifying the imaging parameters according to GS subgroups, applying targeted MRI/US fusion biopsy. Although MRI-targeted biopsies are increasingly being applied in clinical practice, in our study, we did not conduct a direct comparison with systematic biopsies; thus, we cannot assess their effect on decreasing the number of superfluous procedures. Future prospective studies incorporating both targeted and systematic biopsy data may provide stronger evidence to support changes in clinical practice.

## Figures and Tables

**Table 1:** Characteristics of the study population.

Parameter	Values
Number of patients	120
Age (years)	63.35 ± 7.6 (35-80)
PSA (ng/mL)	13.23 ± 18.4
Prostate Volume (mL)	57.12 ± 30
Prostate Density (ng/mL/cc)	0.27 ± 0.33
Ethnicity	100% Turkish origin

**Abbreviations:** PSA: Prostate-Specific Antigen, PD: Prostate Density.

**Table 2:** Comparison of Age, PSA, PI-RADS, and multiparametric magnetic resonance imaging findings according to GS groups.

**Parameter**	**Benign Target(n=138)**	**GS 3+4(n=35)**	**GS >4+3(n=29)**	***p-*value**
Age (years)	62.23 ± 7.9	64.49 ± 6.2	67.03 ± 7.1	>0.02
PSA (ng/mL)	11.04 ± 16.0	11.34 ± 14.0	31.69 ± 31.0	0.000
PSA <10 / >10 (n)	100 / 38	23 / 12	11 / 18	0.002
Prostate Volume (mL)	60.59 ± 31.0	48.48 ± 32.0	46.82 ± 21.0	0.000
Prostate Density (ng/mL/cc)	0.19 ± 0.2	0.28 ± 0.2	0.71 ± 0.5	0.000
PD <0.15 / >0.15 (n)	71 / 67	9 / 26	3 / 26	0.000
Metastatic LN (absent/present)	138 / 0	35 / 0	20 / 9	0.000
Neurovascular Bundle Invasion (no/yes)	138 / 0	35 / 0	20 / 9	0.000
PI-RADS Score (2 / 3 / 4 / 5)	3 / 112 / 22 / 1	0 / 9 / 25 / 1	1 / 2 / 13 / 13	0.000
ADC Mean (mm^2^/s)	0.93 ± 0.13	0.75 ± 0.08	0.62 ± 0.09	<0.05
Lesion Location (PZ / TZ)	118 / 20	32 / 3	26 / 3	>0.05

**Abbreviations:** GS: Gleason Score, PSA: Prostate-Specific Antigen, PD: Prostate Density, PZ: Peripheral Zone, TZ: Transitional Zone, PI-RADS: Prostate Imaging Reporting and Data System, ADC: Apparent Diffusion Coefficient, LN: Lymph Node. **Note:** P-values are based on chi-square test and one-way ANOVA comparisons across groups.

**Table 3:** Pearson correlation analysis of Age, PSA, PI-RADS, and multiparametric magnetic resonance imaging findings.

		**Age**	**PSA**	**Prostate volume**	**PD**	**PI-RADS**	**ADC**
**Age**	r		0.150	0.130	0.138	0.205	-0.156
	p*		0.034	0.065	0.050	0.003	0.027
**PSA**	r	0.150		0.183	0.790	0.543	-0.276
	p*	0.034		0.009	0.000	0.003	0.000
**Prostate volume**	r	0.130	0.183		-0.214	-0.405	0.175
	p*	0.065	0.009		0.002	0.33	0.013
**PD**	r	0.138	0.790	-0.214		0.744	-0.428
	p*	0.050	0.000	0.002		0.000	0.000
**PI-RADS**	r	0.205	0.294	-0.110	0.431		-0.562
	p*	0.003	0.000	0.120	0.000		0.000
**ADC**	r	-0.156	-0.276	0.175	-0.428	-0.562	
	p*	0.027	0.000	0.013	0.000	0.000	

**Abbreviations:** PSA: Prostate Specific Antigen, PD: Prostate Density, PIRADS: Prostate Imaging Reporting and Data System, ADC: Apparent Diffusion Coefficient. **Note:**Values represent Pearson correlation coefficients (r) with corresponding p-values in parentheses (*). Correlations with *p* < 0.05 are considered statistically significant.

**Table 4:** ROC analysis of ADC values (mm^2^/s) for predicting low and high Gleason score prostate cancer.

**Parameter**	**Low GS Malignant Targets (GS 3+4)**	**High GS Malignant Targets (GS >4+3)**
AUC (95% CI)	0.920 (0.874–0.954), *p* < 0.0001	0.947 (0.906–0.973), *p* < 0.0001
Optimal ADC Cut-off	≤ 0.78 mm^2^/sec	≤ 0.70 mm^2^/sec
Sensitivity (%)(95% CI)	79.69	86.21
Specificity (%)(95% CI)	92.03	92.49
PPV (%)(95% CI)	82.25	65.81
NPV (%)(95% CI)	85.71	97.56

**Abbreviations:** ADC: Apparent Diffusion Coefficient, AUC: Area Under the Curve, ROC: Receiver Operating Characteristic, GS: Gleason Score, PPV: Positive Predictive Value, NPV: Negative Predictive Value, CI: Confidence Interval. **Note:** Values indicate diagnostic performance of ADC in detecting low-grade (GS 3+4) and high-grade (GS >4+3) prostate cancer. ROC analysis yielded statistically significant AUC values for both groups (*p* < 0.0001).

**Table 5:** Comparison of anatomical localization of target lesions with histopathological findings.

**Zone / Location**	**Benign (n=138)**	**GS 3+4 (n=35)**	**GS >4+3 (n=29)**	**Total (%)**
**Peripheral Zone (n = 176)**				
Right base	16 (76.2%)	2 (9.5%)	3 (14.3%)	21 (100%)
Right mid	34 (55.7%)	13 (21.3%)	14 (23.0%)	61 (100%)
Right apex	11 (61.1%)	6 (33.3%)	1 (5.6%)	18 (100%)
Left base	9 (69.2%)	3 (23.1%)	1 (7.7%)	13 (100%)
Left mid	32 (76.2%)	5 (11.9%)	5 (11.9%)	42 (100%)
Left apex	16 (76.2%)	3 (14.3%)	2 (9.5%)	21 (100%)
**Transitional Zone (n = 26)**				
Right base	0	0	0	0
Right mid	8 (88.9%)	1 (11.1%)	0	9 (100%)
Right apex	1 (25.0%)	2 (50.0%)	1 (25.0%)	4 (100%)
Left base	2 (100%)	0	0	2 (100%)
Left mid	5 (83.3%)	0	1 (16.7%)	6 (100%)
Left apex	4 (80.0%)	0	1 (20.0%)	5 (100%)

**Abbreviations:** GS: Gleason Score. Right / Left base, mid, apex: Indicates anatomical subregions of the prostate. Peripheral *vs*. Transitional zone: Based on MRI zonal anatomy

**Table 6:** Histopathological results of target lesions and biopsy positivity rates.

**Lesion Region**	**Total Core Count**	**Positive Core Count**	**Positivity Rate (%)**	**Benign**	**GS 3+4**	**GS >4+3**
Peripheral zone – Right mid	61	27	44.3%	34	13	14
Peripheral zone – Left mid	42	10	23.8%	32	5	5
Peripheral zone – Right apex	18	7	38.9%	11	6	1
Transitional zone – Right mid	9	1	11.1%	8	1	0
Transitional zone – Left apex	5	1	20.0%	4	0	1
**Total**	**202**	**81**	**40.1%**	**138**	**35**	**29**

**Table 7:** Distribution of lesions, malignancy, and biopsy core positivity according to PI-RADS scores.

**PI-RADS Score**	**Benign Target (n)**	**GS 3+4 (n)**	**GS >4+3 (n)**	**Total Lesions (n)**	**Malignant Lesions (n, %)**
2	3	0	1	4	1 (25.0%)
3	112	9	2	123	11 (8.9%)
4	22	25	13	60	38 (63.3%)
5	1	1	13	15	14 (93.3%)
**Total**	138	35	29	202	64 (31.7%)

Percentages are based on the number of positive lesions within each PI-RADS category.

## Data Availability

The data of current study are available from corresponding author, [M.H.S], on a reasonable request.

## LIST OF ABBREVIATIONS

ADC: Apparent Diffusion Coefficient
AUC: Area Under the Curve
DWI: Diffusion-Weighted Imaging
GS: Gleason Score
MP-MRI: Multiparametric Magnetic Resonance Imaging
MRI: Magnetic Resonance Imaging
PD: Prostate Density
PI-RADS: Prostate Imaging Reporting and Data System
PSA: Prostate-Specific Antigen
ROC: Receiver Operating Characteristic
ROI: Region of Interest
SD: Standard Deviation
T: Tesla
TE: Echo Time
TR: Repetition Time
TRUS: Transrectal Ultrasound
US: Ultrasound

## STANDARDS OF REPORTING

STROBE guidelines were followed.

## References

[1] Ferlay J., Colombet M., Soerjomataram I., Mathers C., Parkin D.M., Piñeros M., Znaor A., Bray F. (2019). Estimating the global cancer incidence and mortality in 2018: GLOBOCAN sources and methods.. Int. J. Cancer.

[2] Gordetsky J.B., Ullman D., Schultz L., Porter K.K., del Carmen Rodriguez Pena M., Calderone C.E., Nix J.W., Ullman M., Bae S., Rais-Bahrami S. (2019). Histologic findings associated with false-positive multiparametric magnetic resonance imaging performed for prostate cancer detection.. Hum. Pathol..

[3] Falagario U.G., Pellegrino F., Fanelli A. (2024). Prostate cancer detection and complications of MRI-targeted prostate biopsy using cognitive registration, software-assisted image fusion or in-bore guidance: A systematic review and meta-analysis of comparative studies.. Prostate Cancer Prostatic Dis..

[4] Sklinda K., Mruk B., Walecki J. (2020). Active Surveillance of Prostate Cancer Using Multiparametric Magnetic Resonance Imaging: A Review of the Current Role and Future Perspectives.. Med. Sci. Monit..

[5] Quentin M., Boschheidgen M., Radtke J.P., Spohn F., Ullrich T., Drewes L., Valentin B., Lakes J., Al-Monajjed, Arsov C., Esposito I., Albers P., Antoch G., Schimmöller L. (2024). MRI in-bore biopsy following MRI/US fusion-guided biopsy in patients with persistent suspicion of clinically significant prostate cancer.. Eur. J. Radiol..

[6] Lucarelli N.M., Villanova I., Maggialetti N., Greco S., Tarantino F., Russo R., Trabucco S.M.R., Stabile Ianora A.A., Scardapane A. (2023). Quantitative ADC: An additional tool in the evaluation of prostate cancer?. J. Pers. Med..

[7] De Cobelli F., Ravelli S., Esposito A., Giganti F., Gallina A., Montorsi F., Del Maschio A. (2015). Apparent diffusion coefficient value and ratio as noninvasive potential biomarkers to predict prostate cancer grading: comparison with prostate biopsy and radical prostatectomy specimen.. AJR Am. J. Roentgenol..

[8] Ericson K.J., Wu S.S., Lundy S.D., Thomas L.J., Klein E.A., McKenney J.K. (2020). Diagnostic accuracy of prostate biopsy for detecting cribriform Gleason pattern 4 carcinoma and intraductal carcinoma in paired radical prostatectomy specimens: implications for active surveillance.. J. Urol..

[9] Sonn G.A., Natarajan S., Margolis D.J.A., MacAiran M., Lieu P., Huang J., Dorey F.J., Marks L.S. (2013). Targeted biopsy in the detection of prostate cancer using an office based magnetic resonance ultrasound fusion device.. J. Urol..

[10] Gündoğdu E., Emekli E. (2021). Evaluation of prostate volume in mpMRI: comparison of the recommendations of PI-RADS v2 and PI-RADS v2.1.. Diagn. Interv. Radiol..

[11] Omri N., Kamil M., Alexander K., Alexander K., Edmond S., Ariel Z., David K., Gilad A.E., Azik H. (2020). Association between PSA density and pathologically significant prostate cancer: The impact of prostate volume.. Prostate.

[12] Dell’Oglio P., Stabile A., Soligo M., Brembilla G., Esposito A., Gandaglia G., Fossati N., Bravi C.A., Dehò F., De Cobelli F., Montorsi F., Karnes R.J., Briganti A. (2020). There is no way to avoid systematic prostate biopsies in addition to multiparametric magnetic resonance imaging targeted biopsies.. Eur. Urol. Oncol..

[13] Stabile A., Dell’Oglio P., Gandaglia G., Fossati N., Brembilla G., Cristel G., Dehò F., Scattoni V., Maga T., Losa A., Gaboardi F., Cardone G., Esposito A., De Cobelli F., Del Maschio A., Montorsi F., Briganti A. (2018). Not all multiparametric magnetic resonance imaging–targeted biopsies are equal: The impact of the type of approach and operator expertise on the detection of clinically significant prostate cancer.. Eur. Urol. Oncol..

[14] Clark R., Vesprini D., Narod S.A. (2022). The effect of age on prostate cancer survival.. Cancers.

[15] Mazzone E., Stabile A., Sorce G., Pellegrino F., Barletta F., Motterle G., Scuderi S., Cirulli G.O., Cucchiara V., Brembilla G., Esposito A., Gandaglia G., Fossati N., De Cobelli F., Montorsi F., Karnes R.J., Guccini I., Briganti A. (2021). Age and gleason score upgrading between prostate biopsy and radical prostatectomy: Is this still true in the multiparametric resonance imaging era?. Urol. Oncol..

[16] David M, Leslie S. (2024). Prostate-Specific Antigen.. StatPearls.

[17] Kim T.H., Kim C.K., Park B.K., Jeon H.G., Jeong B.C., Seo S.I., Lee H.M., Choi H.Y., Jeon S.S. (2016). Relationship between Gleason score and apparent diffusion coefficients of diffusion-weighted magnetic resonance imaging in prostate cancer patients.. Can. Urol. Assoc. J..

[18] Han L., He G., Mei Y., Yu Q., Zhao M., Luo F., Cheng G., Liang W. (2022). Combining Magnetic Resonance Diffusion-Weighted Imaging with Prostate-Specific Antigen to Differentiate Between Malignant and Benign Prostate Lesions.. Med. Sci. Monit..

[19] Dai Z., Liu Y., Huangfu Z., Wang L., Liu Z. (2021). Magnetic Resonance Imaging (MRI)-Targeted Biopsy in Patients with Prostate-Specific Antigen (PSA) Levels <20 ng/mL: A Single-Center Study in Northeastern China.. Med. Sci. Monit..

[20] Turkbey B., Mani H., Aras O., Rastinehad A.R., Shah V., Bernardo M., Pohida T., Daar D., Benjamin C., McKinney Y.L., Linehan W.M., Wood B.J., Merino M.J., Choyke P.L., Pinto P.A. (2012). Correlation of magnetic resonance imaging tumor volume with histopathology.. J. Urol..

[21] deSouza N.M., Riches S.F., VanAs N.J., Morgan V.A., Ashley S.A., Fisher C., Payne G.S., Parker C. (2008). Diffusion-weighted magnetic resonance imaging: a potential non-invasive marker of tumour aggressiveness in localized prostate cancer.. Clin. Radiol..

[22] Tan C.H., Wei W., Johnson V., Kundra V. (2012). Diffusion-weighted MRI in the detection of prostate cancer: meta-analysis.. AJR Am. J. Roentgenol..

[23] Hambrock T., Somford D.M., Huisman H.J., van Oort I.M., Witjes J.A., Hulsbergen-van de Kaa C.A., Scheenen T., Barentsz J.O. (2011). Relationship between apparent diffusion coefficients at 3.0-T MR imaging and Gleason grade in peripheral zone prostate cancer.. Radiology.

[24] Salami S.S., Ben-Levi E., Yaskiv O., Turkbey B., Villani R., Rastinehad A.R. (2017). Risk stratification of prostate cancer utilizing apparent diffusion coefficient value and lesion volume on multiparametric MRI.. J. Magn. Reson. Imaging.

[25] Kumar V., Jagannathan N.R., Kumar R., Thulkar S., Gupta S.D., Dwivedi S.N., Hemal A.K., Gupta N.P. (2007). Apparent diffusion coefficient of the prostate in men prior to biopsy: determination of a cut-off value to predict malignancy of the peripheral zone.. NMR Biomed..

[26] Girometti R., Bazzocchi M., Como G., Brondani G., Del Pin M., Frea B., Martinez G., Zuiani C. (2012). Negative predictive value for cancer in patients with “Gray-Zone” PSA level and prior negative biopsy: Preliminary results with multiparametric 3.0 tesla MR.. J. Magn. Reson. Imaging.

[27] Isebaert S., Van den Bergh L., Haustermans K., Joniau S., Lerut E., De Wever L., De Keyzer F., Budiharto T., Slagmolen P., Van Poppel H., Oyen R. (2013). Multiparametric MRI for prostate cancer localization in correlation to whole-mount histopathology.. J. Magn. Reson. Imaging.

[28] Wu L.M., Xu J.R., Ye Y.Q., Lu Q., Hu J.N. (2012). The clinical value of diffusion-weighted imaging in combination with T2-weighted imaging in diagnosing prostate carcinoma: a systematic review and meta-analysis.. AJR Am. J. Roentgenol..

[29] Lodeta B., Trkulja V., Kolroser-Sarmiento G., Jozipovic D., Salmhofer A., Augustin H. (2021). Systematic biopsy should not be omitted in the era of combined magnetic resonance imaging/ultrasound fusion-guided biopsies of the prostate.. Int. Urol. Nephrol..

[30] Ploussard G., Borgmann H., Briganti A., de Visschere P., Fütterer J.J., Gandaglia G., Heidegger I., Kretschmer A., Mathieu R., Ost P., Sooriakumaran P., Surcel C., Tilki D., Tsaur I., Valerio M., van den Bergh R., EAU-YAU Prostate Cancer Working Group (2019). Positive pre-biopsy MRI: are systematic biopsies still useful in addition to targeted biopsies?. World J. Urol..

[31] Bengtsson J., Thimansson E., Baubeta E., Zackrisson S., Sundgren P.C., Bjartell A., Flondell-Sité D. (2023). Correlation between ADC, ADC ratio, and Gleason Grade group in prostate cancer patients undergoing radical prostatectomy: Retrospective multicenter study with different MRI scanners.. Front. Oncol..

